# Efficacy of adjuvant radiotherapy for atypical and anaplastic meningioma

**DOI:** 10.1002/cam4.1531

**Published:** 2019-01-25

**Authors:** Hongda Zhu, Wenya Linda Bi, Ayal Aizer, Lingyang Hua, Mi Tian, Jiaojiao Den, Hailiang Tang, Hong Chen, Yin Wang, Ying Mao, Ian F. Dunn, Qing Xie, Ye Gong

**Affiliations:** ^1^ Department of Neurosurgery Huashan Hospital Shanghai Medical College Fudan University Shanghai China; ^2^ Department of Neurosurgery Cener for Skull Base and Pituitary Surgery Brigham and Women's Hospital Harvard Medical School Boston MA USA; ^3^ Department of Radiation Oncology Brigham and Women's Hospital Harvard Medical School Boston MA USA; ^4^ Department of Neuropathology Huashan Hospital Fudan University Shanghai China; ^5^ Department of Critical Care Medicine Huashan Hospital Fudan University Shanghai China

**Keywords:** anaplastic meningioma, atypical meningioma, overall survival, progression‐free survival, radiation therapy

## Abstract

The effect of adjuvant radiotherapy in management for high‐grade meningiomas, especially atypical meningiomas, remains controversial. We aimed to explore the role of adjuvant radiotherapy in this population. A total of 162 adults with high‐grade meningiomas (99 atypical meningiomas and 63 anaplastic meningiomas) were treated from 2003 to 2008 at Huashan Hospital. One hundred and seventeen patients presented with primary and 45 with recurrent disease. One hundred and fifteen patients (70.9%) were treated with adjuvant radiotherapy after surgical resection. The median follow‐up was 76.5 months (range 1‐142 months). Kaplan‐Meier survival curve and Cox proportional hazards modeling were used for analyses. Adjuvant radiotherapy was associated with prolonged progression‐free survival (PFS) and overall survival (OS) in patients with newly diagnosed anaplastic meningiomas irrespective of extent of resection (PFS,* P* = .001; OS,* P* = .003). Gross total resection was the only independent prognostic factor for those with newly diagnosed atypical meningiomas (PFS,* P* < .001; OS,* P* = .012). A survival benefit for adjuvant radiation was also found in subgroup analysis of patients with high‐grade meningiomas who underwent subtotal resection (PFS,* P* = .023; OS,* P* = .013). Among recurrent high‐grade meningiomas, radiotherapy offered no statistically significant improvement in either PFS or OS. Adjuvant radiotherapy is associated with improved survival in patients with newly diagnosed anaplastic meningiomas and those high‐grade meningiomas following subtotal resection. However, there was no significant correlation identified between postoperative radiation and outcome for recurrent high‐grade meningiomas. Future prospective randomized trials may help clarify the optimal tailored treatment for patients with high‐grade meningioma.

## 1 INTRODUCTION

Meningioma account for a third of the primary tumors of the central nervous system.^1^ The majority of meningiomas are histologically benign and may be surgically curable. However, atypical and anaplastic meningiomas, as classified by the World Health Organization (WHO), are prone to recurrence. The five‐year progression‐free survival (PFS) and overall survival (OS) rate for atypical meningioma are 48‐68% and 78‐91%, respectively, and 8‐61% and 35‐79% for anaplastic meningioma.^2^, ^3^, ^4^, ^5^, ^6^, ^7^, ^8^, ^9^

While the administration of adjuvant radiotherapy for high‐grade meningioma has been shown to be effective to prolong progression‐free survival,^4^, ^6^, ^7^, ^8^ its effect on overall survival remains less clear.^3^, ^4^, ^5^, ^6^, ^7^, ^8^, ^9^, ^10^, ^11^, ^12^ Furthermore, no consensus exists on the role of adjuvant radiation in meningiomas on recurrence or following gross total resection (GTR).^3^, ^10^, ^11^, ^12^, ^13^ Thus, a standard of care for nonbenign meningioma remains challenging to define.

We retrospectively analyzed 162 adults with high‐grade meningiomas from a single institution to determine the influence of clinical features, treatment status, tumor location, extent of surgical resection, and administration of adjuvant radiation, on outcome.

## 2 MATERIALS AND METHODS

### 2.1 Patient population

We reviewed the clinical records of 99 adults with atypical meningioma who were operated on from 2005 to 2008 and 63 adults with anaplastic meningioma from 2003 to 2008 in the Department of Neurosurgery, Huashan Hospital of Fudan University, Shanghai, China. The year 2008 was chosen as the last year of study to allow for adequate follow‐up of patients. Informed consent was obtained in compliance with the Human Subjects Institutional Review Board at Huashan Hospital.

In general, adjuvant radiation was recommended to both atypical and anaplastic meningioma patients, regardless of GTR or STR. And the final decision was made based on the negotiation with the relatives of patients. Electronic portal imaging device (MV‐EPID) and cone beam CT (kV‐CBCT) (Elekta Synergy system, Stockholm, Sweden) were used for radiotherapy treatment planning. In total, 115 patients (70.9%) received postoperative radiation. Among these, 103 (89.6%) were treated with external beam radiotherapy (EBRT) on tumor bed in 2.0 Gy daily fractions with 1‐ to 2‐cm clinical target volume (CTV) along dural surfaces and 3‐ to 5‐mm planning target volume (PTV) (mean dose 54.3 ± 5.2 Gy, range 30‐63 Gy); 12 (10.4%) were treated with stereotactic radiosurgery (SRS) with prescription dose 14.0 Gy at 50% (28.0 Gy at 100%) and median isodose of 88% (range 85‐92). Ten anaplastic meningioma patients were treated with concomitant chemotherapy (nimustine, semustine, or temozolomide).

### 2.2 Pathology review

Pathologic diagnoses for all patients were reviewed by two senior neuropathologists (Dr. H Chen and Dr. HX Chen). The tumors were classified according to the 2000 WHO grading system criteria.^14^ Atypical meningiomas are defined by presence of 4‐19 mitoses per 10 high‐power fields (HPF) or three of the following five features: increased cellularity, high nuclear to cytoplasmic ratio, prominent nucleoli, loss of architectural pattern, and spontaneous necrosis not due to embolization. Anaplastic meningiomas are defined by presence of 20 or greater mitoses per 10 HPF, or malignant cytological features (i.e., resemblance to sarcoma or carcinoma). Brain invasion was noted, but was not considered a diagnostic criterion for atypical grade.

### 2.3 Clinical outcomes and statistical analysis

Prognostic factors considered for statistical analysis included: age (<60 years or ≥60 years), gender (male or female), Karnofsky Performance Status (KPS) (<80 or ≥80), Simpson grade of resection (grade I‐II vs grade III‐IV), treatment status (newly diagnosed vs recurrent), tumor location (skull base or non‐skull base), MIB‐1 labeling index (<5% or ≥5%), and adjuvant radiation. The extent of resection was graded according to the Simpson classification: Simpson grade I‐II was considered gross total resection (GTR), and Simpson grade III‐IV resection was considered subtotal resection (STR) for purposes of analyses.^15^ Tumor location was classified as skull base, convexity, falx/parasagittal, intraventricular, or other. Progression‐free survival (PFS) was defined as time from diagnosis to the detection of tumor regrowth on follow‐up MRI prompted in some cases or on progression or recurrence of symptoms. The Acute and Late Radiation Morbidity Scoring Criteria of the Radiation Therapy Oncology Group (RTOG) was applied to evaluate radiotherapy‐related toxicity.

Categorical variables were compared with the chi‐square test, and continuous variables with the independent‐samples Student's *t*‐test (data with normal distribution) or Mann‐Whitney U‐test (data with skewed distribution). Continuous data were expressed as the mean ± standard deviation (SD). Survival curves were generated using the Kaplan‐Meier method; differences were assessed by log‐rank tests. Cox proportional hazards model was used to analyze possible prognostic factors. All analyses were performed using Statistical Package for Social Sciences (SPSS; Version 20.0, Chicago, IL, USA). Data were considered to be significant when *P* < .05.

## 3 RESULTS

### 3.1 Clinical and pathological characteristics

One hundred and sixty‐two adults (79 females, 83 males) were identified with atypical or anaplastic meningiomas, with mean age of 51.2 ± 14.2 years (range 18‐80 years). The male to female ratio was 1:1.06 for atypical and 1.25:1 for anaplastic meningioma. Median follow‐up was 76.5 months (range 1‐142 months). The mean MIB‐1 labeling index for atypical and anaplastic meningioma was 4.8 ± 5.2% and 9.8 ± 13.0%, respectively (*P* = .005). Clinicopathological characteristics for patients who did and did not receive adjuvant radiation are summarized in Table 1. No significant differences were observed in patient age, gender, treatment status (newly diagnosed or recurrent), tumor location, extent of resection, and MIB‐1 proliferative index between these two groups. Patients who had higher preoperative KPS score were more prone to receive radiation therapy (*P* = .004).

**Table 1:** Clinicopathological characteristics of meningioma patients who did and did not receive radiation

Feature	Radiation	No radiation	*P*
Patients, *n*	115	47	
Age, mean±SD, year (range)	50.0 ± 13.4 (18‐80)	54.1 ± 15.8 (19‐79)	.06
Gender, *n* (%)			
Male	56 (49)	27 (57)	.31
Female	59 (51)	20 (43)	
Initial status, *n* (%)			
*De novo*	85 (74)	32 (68)	.45
Recurrent	30 (26)	15 (32)	
KPS			
≥80	75 (65)	19 (40)	.004^a^
<80	40 (35)	28 (60)	
Tumor location, *n* (%)			
Skull base	25 (22)	12 (26)	.60
Non‐skull base	90 (78)	35 (74)	
Extent of resection, *n* (%)			
GTR	91 (79)	36 (77)	.72
STR	24 (21)	11 (23)	
Subtype, *n* (%)			
Atypical	72 (63)	27 (57)	.54
Anaplastic	43 (37)	20 (43)	
MIB‐1 labeling index			
≥5%	51 (44)	22 (47)	.78
<5%	64 (56)	25 (53)	

KPS, Karnofsky performance status; EBRT, external beam radiation therapy; GTR, gross total resection; STR, subtotal resection; *n*, number; SD, standard deviation.

a *P* < .05 considered statistically significant.

Among the 162 patients, 117 (72.2%) were newly diagnosed meningiomas while the remaining 45 patients presented with recurrent meningioma following prior surgical resection. About 60% (12/20) of recurrent atypical meningiomas and 72% (18/25) of recurrent anaplastic tumors resulted from malignant progression from a lower histological grade. A total of 115 patients (70.9%) were treated with adjuvant radiotherapy after surgical resection.

### 3.2 Patients with newly diagnosed high‐grade meningiomas have longer survival than recurrent meningiomas

**Figure 1:**
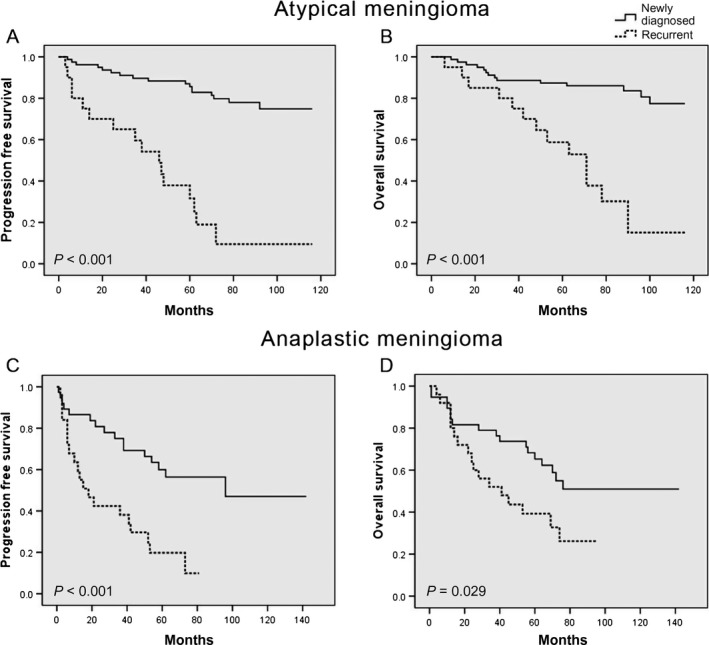
Impact of primary vs recurrent tumor status on survival. Kaplan‐Meier curves for (A) progression‐free survival and (B) overall survival in newly diagnosed vs recurrent atypical meningioma; and (C) progression‐free survival and (D) overall survival in newly diagnosed vs recurrent anaplastic meningioma

We first established the baseline survival rates for atypical and anaplastic meningioma patients in our cohort. Consistent with the literature, the mean PFS and OS for patients with newly diagnosed atypical meningioma were statistically longer than those with a newly diagnosed anaplastic meningioma (PFS, 99.0 ± 3.8 months vs 88.6 ± 10.0 months, *P* = .004; OS, 101.9 ± 3.6 months vs 91.7 ± 9.2 months, *P* = .001), and trended to be longer in patients with recurrent atypical meningioma compared to recurrent anaplastic meningiomas (PFS, 44.6 ± 7.7 months vs 30.7 ± 5.7 months, *P* = .339; OS, 64.5 ± 7.9 months vs 48.5 ± 6.9 months, *P* = .306). Subsequent tumor recurrence occurred in 80% (36/45) of patients who presented with recurrent disease and 28.2% (33/117) of patients with newly diagnosed meningiomas. Log‐rank comparison of Kaplan‐Meier curves revealed that patients with recurrent disease have shorter PFS and OS compared to those with newly diagnosed tumors for both atypical (PFS, HR 7.321; 95% CI, 3.590‐14.932; *P* < .001; OS, HR 5.716; 95% CI, 2.616‐12.493; *P* < .001) and anaplastic meningiomas (PFS, HR 3.359; 95% CI, 1.687‐6.690; *P* < .001; OS, HR 2.093; 95% CI, 1.059‐4.137; *P* = .029) (Figure 1).

### 3.3 Impact of adjuvant radiation on survival

We next examined the impact of adjuvant radiation on survival in high‐grade meningioma patients, as stratified by extent of resection and treatment status on presentation.

**Figure 2:**
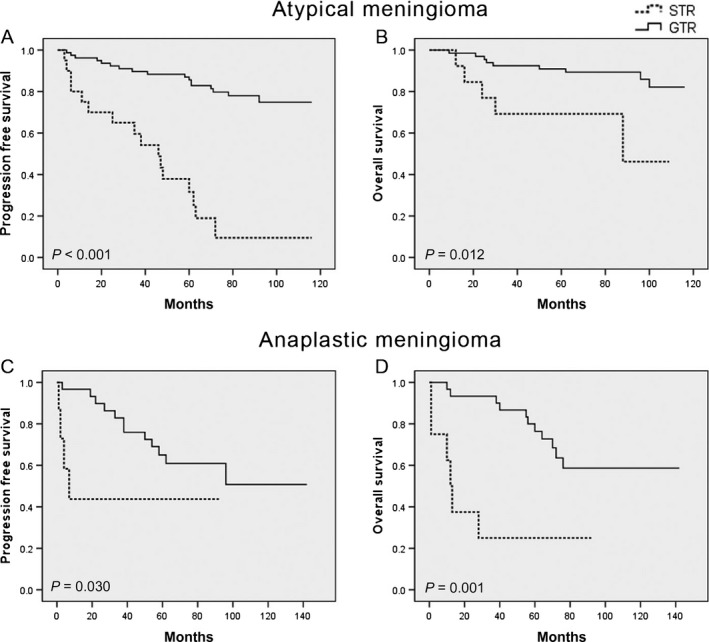
Impact of extent of resection on survival in newly diagnosed subgroups. Kaplan‐Meier curves for (A) progression‐free survival and (B) overall survival in newly diagnosed atypical meningiomas following GTR or STR; and (C) progression‐free survival and (D) overall survival in newly diagnosed anaplastic meningiomas following GTR or STR

Simpson grade I‐II resection was achieved in 127 tumors (78.4%) with the remaining 35 cases (21.6%) undergoing Simpson grade III‐IV resection. Among newly diagnosed tumors, with 79 atypical and 38 anaplastic meningiomas, Kaplan‐Meier analysis indicated that there was a significant correlation between GTR and improved outcome for both atypical (PFS, HR 0.109; 95% CI, 0.041‐0.286; *P* < .001; OS, HR 0.266; 95% CI, 0.088‐0.803; *P* = .012) and anaplastic meningiomas (PFS, HR 0.295; 95% CI, 0.092‐0.951; *P* = .030; OS, HR 0.191; 95% CI, 0.067‐0.542; *P* = .001) (Figure 2).

**Figure 3:**
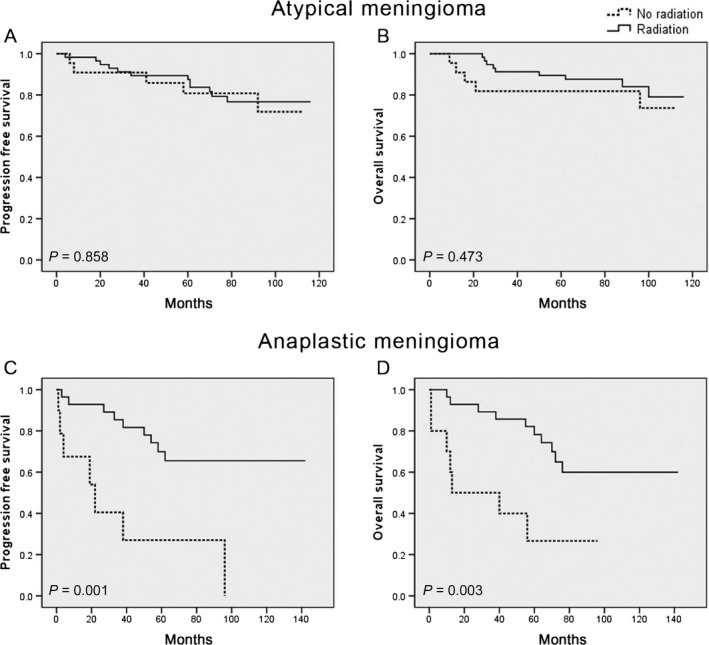
Impact of adjuvant radiation on survival in newly diagnosed subgroups. Kaplan‐Meier curves for (A) progression‐free survival and (B) overall survival in newly diagnosed atypical meningiomas following adjuvant radiation; and (C) progression‐free survival and (D) overall survival in newly diagnosed anaplastic meningiomas following adjuvant radiation

**Figure 4:**
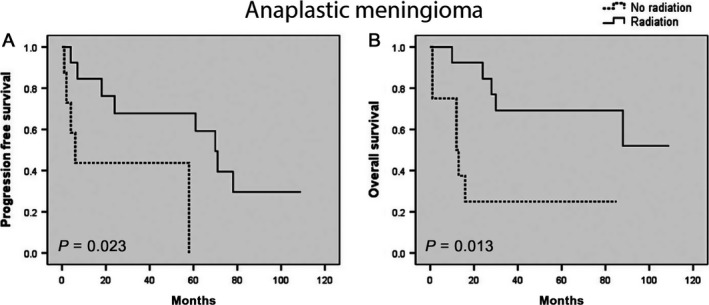
Kaplan‐Meier curve for (A) progression‐free survival and (B) overall survival in newly diagnosed anaplastic meningiomas with STR following adjuvant radiation

Among newly diagnosed meningioma patients with gross total resection (defined as Simpson grade I‐II), the use of adjuvant radiation was associated with longer PFS (HR 0.182; 95%, 0.051‐0.612; *P* = .001) and OS (HR 0.193; 95% CI, 0.058‐0.711; *P* = .003) for anaplastic meningiomas, but had no statistically significant association with either local control or overall survival for atypical meningiomas in this cohort (PFS, HR 0.695; 95% CI, 0.166‐2.918; *P* = .858; OS, HR 0.646; 95% CI, 0.211‐3.402; *P* = .473) (Figure 3). Adjuvant radiation was also associated with improved PFS (HR 0.238; 95% CI, 0.061‐0.815; *P* = .023) and OS (HR 0.223; 95% CI, 0.061‐0.820; *P* = .013) in newly diagnosed atypical (n = 13) and anaplastic meningioma patients (n = 5) who underwent subtotal resection (Figure 4).

In comparison, among 45 patients with recurrent meningioma (20 atypical, 25 anaplastic), GTR was achieved in 70% (14/20) atypical cases and 64% (16/25) in anaplastic cases. Administration of adjuvant radiation was not associated with a significant difference in progression‐free (HR 0.555; 95% CI, 0.278‐1.108; *P* = .086) or overall survival (HR 0.654; 95% CI, 0.309‐1.383; *P* = .259), independent of the extent of resection.

Acute toxicities occurred in 18 patients including: grade 1 skin reaction (n = 9), grade 2 hearing deterioration (n = 1), grade 1‐2 upper gastro‐intestinal toxicity (n = 6), and grade 1‐3 cognitive disturbance (n = 8). During the follow‐up period, no adverse radiation effect was observed on any patients.

### 3.4 Prognostic factors influencing PFS and OS

We examined the impact of covariates on survival by performing multivariate analysis and observed that increased extent of resection was significantly associated with prolonged PFS (HR 0.071; 95% CI, 0.024‐0.210; *P* < .001) and OS (HR 0.216; 95% CI, 0.067‐0.695; *P* = .010) among newly diagnosed atypical meningioma (Table 2). Among the 38 newly diagnosed anaplastic meningiomas, multivariate analysis identified adjuvant radiation as the only independent factor affecting PFS, while extent of resection and preoperative KPS were significant prognostic factors for OS (Table 2).

**Table 2:** Multivariate analysis of prognostic factors in patients with *de novo* atypical and anaplastic meningiomas

Variable	*De novo* atypical meningiomas				*De novo* anaplastic meningiomas			
	Progression‐free survival		Overall survival		Progression‐free survival		Overall survival	
	*P*	HR (95%CI)	*P*	HR (95%CI)	*P*	HR (95%CI)	*P*	HR (95%CI)
KPS (≥80/<80)							.035^a^	0.335 (0.121‐0.927)
Extent of resection (GTR/STR)	<.001^a^	0.071 (0.024‐0.210)	.010^a^	0.216 (0.067‐0.695)			.002^a^	0.177 (0.059‐0.529)
Radiation (Yes/No)					.002^a^	0.198 (0.072‐0.549)		

KPS, Karnofsky performance status; OR, odds ratio; CI, confidence interval; GTR, gross total resection; STR, subtotal resection.

a *P* < .05 considered statistically significant.

In the recurrent group, nonskull base tumor location (HR 0.219; 95% CI, 0.068‐0.706; *P* = .011), greater extent of resection (HR 0.219; 95% CI, 0.068‐0.706; *P* = .011), and lower MIB‐1 labeling index (HR 0.225; 95% CI, 0.068‐0.743; *P* = .014) were associated with longer PFS for atypical meningiomas, while no factor demonstrated prognostic significance for OS. Cox regression analysis did not identify any factors with a significant correlation to either PFS or OS for recurrent anaplastic meningiomas.

## 4 DISCUSSION

We investigated the prognostic factors associated with clinical outcome in high‐grade meningioma patients following surgery and adjuvant radiation. We observed that adjuvant radiotherapy was correlated with improved PFS and OS for patients with newly diagnosed anaplastic meningiomas, and those who underwent a STR of newly diagnosed high‐grade meningiomas.

The role of adjuvant radiotherapy in atypical meningioma with GTR remains controversial.^3^, ^10^, ^12^, ^16^, ^17^ Simpson grade I or II resection without adjuvant radiotherapy has been demonstrated to achieve durable local control and long survival,^3^, ^9^ while others have also advocated rendering adjuvant radiotherapy in patients with atypical meningioma even after GTR.^10^, ^11^ In our study, adjuvant radiotherapy did not associate with improved PFS or OS of patients who received GTR of a newly diagnosed atypical meningioma. In comparison, a recent study with 91 primary atypical meningiomas, using a propensity score model, reported a positive correlation between adjuvant radiation therapy and local control, advocating for its administration in patients following GTR.^11^ As patients in our study received postoperative radiotherapy ranging from 44 to 62 Gy, this intrinsic variance might limit the power of the results, as other publications have demonstrated an association between higher radiation doses and improved clinical outcomes.^18^ Milosevic et al. reported a dose of ≥50 Gy to be strongly associated with improved OS in atypical and anaplastic meningioma patients,^19^ while Hug et al. indicated that the actuarial five‐ and eight‐year local control rates for atypical meningioma were significantly higher with doses ≥60 Gy vs 60 Gy.^20^ In comparison, it is widely accepted that patients with anaplastic meningioma benefit from adjuvant radiotherapy.^4^, ^5^, ^6^, ^7^, ^8^, ^16^, ^17^, ^18^

Notably, the effect of radiation is influenced by the primary or recurrent status of meningiomas. One study of 48 malignant meningiomas from 38 patients noted that adjuvant radiotherapy was associated with a reduced recurrence rate only in patients with primary meningiomas, consistent with the result of our study.^4^ In this series, adjuvant radiotherapy was identified as a strong independent factor for local control in newly diagnosed anaplastic meningioma (*P* = .002), but was not associated with clinical outcome in the recurrent group. Moreover, in patients with recurrent high‐grade meningioma, Cox regression analysis failed to identify any factor with significant association with either PFS or OS. However, the result from another retrospective study suggested better tumor control with the addition of radiation for patients with recurrent high‐grade meningioma.^21^ Thus, a larger cohort or multicenter clinical trial is needed to investigate the effect of radiation in this subgroup.

Adjuvant radiotherapy is generally recommended for patients with primary high‐grade meningioma after incomplete resection,^6^, ^7^, ^10^, ^22^ with isolated reports suggesting that postoperative stereotactic radiosurgery is not associated with a statistically significant PFS benefit for patients with atypical meningioma following STR.^16^ A relatively high GTR rate in this series limited further stratification. On combined analysis of subtotally resected newly diagnosed atypical and anaplastic meningiomas, we found that adjuvant radiotherapy was significantly associated with improvement in both PFS and OS.

Several limitations of our study warrant consideration. This study was retrospective, thus, some biases might exist that are difficult to statistically control. Although we examined specific subgroups to distinguish potential influences of major clinical factors on the outcome, a relatively limited sample size undermined the statistical power. Radiation dosages varied across time, as noted above, and may influence the clinical outcome of patients. Lastly, due to sample size, we clustered Simpson grade categories for extent of resection analyses, which may obscure further heterogeneity in outcome had the data been stratified by distinct Simpson grades.

## 5 CONCLUSION

Our study indicates that adjuvant radiotherapy is associated with improved survival in patients who undergo subtotal resection of a newly diagnosed grade II‐III meningioma and all patients with newly diagnosed anaplastic meningiomas. Future clinical trials may help clarify the role of adjuvant radiotherapy in recurrent high‐grade meningiomas, to better tailor treatment to the individual setting.

## CONFLICT OF INTEREST

The authors declare no conflicts of interest in relation to this research and its publication.
